# A Comparison of a Multi-body Model and 3D Kinematics and EMG ofDouble-leg Circle on Pommel Horse

**DOI:** 10.2478/v10078-012-0005-9

**Published:** 2012-04-03

**Authors:** Jing-guang Qian, Yang Su, Ya-wei Song, Ye Qiang, Songning Zhang

**Affiliations:** 1 Department of Human Sports Science, Nanjing Institute of Physical Education, China.; 2 Department of Kinesiology, Recreation and Sport Studies, The University of Tennessee, USA.

**Keywords:** gymnastics, DLC, kinematics, computer simulation, COG

## Abstract

The purpose of this study was to establish a multi-segment dynamic model in the LifeMOD to examine kinematics of the center of mass and foot, and muscle forces of selected upper extremity muslces during a double-leg circle (DLC) movement on pommel horse in gymnastics and compared with three-dimensional kinematics of the movement and surface electromyographic (sEMG) activity of the muscles. The DLC movement of one elite male gymnast was collected. The three-dimensional (3D) data was imported in the Lifemod to create a full-body human model. A 16-Channel surface electromyography system was used to collect sEMG signals of middle deltoid, biceps brachii, triceps brachii, latissimusdorsi, and pectoralis major. The 3D center of mass and foot displacement showed a good match with the computer simulated results. The muscle force estimations from the model during the four DLC phases were also generally supported by the integrated sEMG results, suggesting that the model was valid. A potential application of this model is to help identify shortcomings of athletes and help establish appropriate training plans errors in the DLC technique during training.

## Introduction

The development of multi-body dynamics system theory has provided a powerful platform for design, analysis and optimization of complex systems. It has also opened new avenues for research in sports (Qian, 2006).Liu used this method to establish a four-segment model to study the standing vertical jump (Liu, 1987). Qian conducted a computer simulation and training study on an innovation of a backward somersault with re-grab on a high bar (Qian, 2006). In 2004, Liu established a 3-segment rigid body model of upper limb with 7 degrees of freedom and a physical model of upper limb to study whip movements using Kane’s dynamics approach (Liu, 2004).

Applications using complicated dynamical systems and computer simulations have gained increased popularity in research and training in gymnastics, diving and other sports. Arampatzis et al. (2002) studied the influence of different mats on foot motion during landing in gymnastics. A multi-segment model was used to investigate optimal compliant-surface in springboard standing jumps (Cheng and Hubbard, 2005). The role of arm swing in compliant-surface jumping for maximizing backward somersault rotations was further examined using a multi-segment model in springboard diving (Cheng and Hubbard, 2008). To optimize the performance of off-road bicycle suspension systems, it was suggested that a dynamic model of the bicycle/rider system would be useful (Wang and Hull, 1997). In addition, Spägele et al. (1999) described an efficient biomechanical model of the human lower limb with the aim of simulating a real human jump movement. Furthermore, a planar eight-segment model with extensor and flexor torque generators at five joints was formulated to examine performance of an elite male high jumper (Wilson et al., 2007).

Lifemod is an advanced multi-body computer simulation software system commonly used in human movement simulation with ADAMS as the dynamics modeling engine. Chiu (1994) used the Lifemod to establish the model of a human head, neck and upper torso. Lifemod was used to set up a cervical model to mimic invivo conditions (de Jongh et al., 2007). Shi et al. (2007) developed a dynamic model of a knee joint after total knee replacement using ADAMS. Song and Zhang (2002) used the ADAMS to simulate the upperarm and forearm with a 2-segment rigid body system with a ball-and-socket articulation between the upperarm and torso and a hinge joint between the forearm and upperarm, and simulated movements of internal and external rotations, abduction and adduction, and flexion and extension. In addition to the modeling of body segments and their dynmics, muscle functions of the human body were simplified as reaction forces supplied to the center of mass. It was demonstrated that the simulation results using the model were rather consistent with the actual motions of the upper limb.

Although there have been several attempts to examine double-leg circles (DLC) on the pommel horse in gymnastics (Baudry et al., 2008; 2009), research on this fundemental movement of pommel horse is still rather limited. Therefore, the purpose of this study was to establish a multi-segment dynamics model in the Lifemod and validate the model by comparing kinematics of the center of mass and foot and muscle forces of selected upper extremity muslces from the model to the three-dimensional (3D) kinematics of the movement and surface electromyographic (sEMG) activity of the muscles during a DLC movement. Due to the exploratory nature of this study, no hypothesis was generated.

## Material and Methods

The experimental 3D and sEMG data of the DLC movement from an elite male gymnast of the gymnastic team in the JiangSu province, China, was collected. An informed consent approved by the research office of the institution was obtained prior to the data collection.

A six-camera 3D motion analysis system (60 Hz, Motion Analysis Co, USA) was used to collect kinematic data of the movement. Thirty five reflective markers were placed in the upper extremity, trunk and lower extremity using a marker set recommended by human modeling and simulation software Lifemod (Biomechanics Research Group, Inc., USA). A lab coordinate system was set up so that the anterioposterior direction is perpendicular to the horse, the mediolateral direction parallel to the horse. A 16-Channel surface electromyography System (1000Hz, TeleMyo 2400R, Noraxon, USA) was used to collect sEMG Signals of middle Deltoid (DT), Biceps brachii (BB), Triceps brachii (TB), Latissimusdorsi (LD), and Pectoralis major (PM), simultaneously with the kinematic data using the Motion Analysis System.

During the data collection, the athlete warmed up for 30 minutes. The skin surface of the muscle was first cleaned with 75% alcohol. The sEMG electrode was attached to the center of each tested muscle. The inter-electrode distance was 20 mm. The athlete then performed two sets of DLC separately with 5 trials (cycles) each and a 15-minute rest was provided between the sets. The sEMG and kinematic data from the 3^rd^ trial were selected for further analyses. The movement cycle was divided into four phases according to whether the body was in double or single arm support on the horse: anterior double arm support (T1), left arm support (T2), posterior double arm (T3), and right arm support (T4). The iEMG values of each muscle in each phase were normalized by the highest iEMG value of each muscle in all four phases of the cycle.

The next part of the study was to use the Lifemod to simulate the movement. The first step was to create a complete human body model using one of several anthropometric databases which included joint sets and muscle groups. The data (EXCEL files) captured from the Motion Analysis capture system was then imported into the Lifemod. The next step was to perform an inverse dynamics analysis. Finally, a forward-dynamics simulation was performed to obtain the kinematic and dynamic data of the movement cycle.

## Results

### COG displacement

The COG displacement from the 3D kinematic and the simulated results were provided in Table 1. The data indicated a perfect match in the mediolateral and vertical directions and a good match in the anteroposterior direction between the 3D and simulated results (Table 1).

***Foot displacement***. The 3D displacements of the foot of the simulated (Figure 1a) and 3D results (Figure 1b) demonstrated similar movement patterns. The origins of the coordinate system for the Lifemod and 3D kinematics were different and therefore the absolute values in those figures could not be directly compared. The displacement patterns for each direction were very similar to the simulated and 3D data except for the vertical displacement. The displacement of the left foot was selected to examine the overall foot movement during the cycle. The 3D and simulated results of the foot displacement showed good matches (Table 1).

***Muscle forces***. The muscle forces of the five selected muscles computed in Lifemod during the movement circle of DLC are shown in Figures 2a and b. First of all, both left and right triceps brachii had the greatest muscle force among the five muscles during the movement cycle. Secondly, the pectoralis major and biceps brachii showed no obvious peak forces, but continuous effort throughout most of the DLC cycle. The deltoid muscle reached the maximum in phase 1 to stabilize the shoulders, then decreased its magnitude until the later part of phase 4 to prepare for the next cycle. Finally, the right latissimusdorsi and triceps brachii reached the peak forces in phase 1 and 4.

***Integrated EMG***. The right and left integrated EMG of the five tested muscles for the four movement phases are presented in Figure 3a and 3b.

## Discussion

It is very important to improve stability of the double-leg circles on pommel horse because of its movement characteristics. Due to the complexity of the movement, research on DLC is relatively sparse in the literature. Grassi et al. (2005) examined the pelvis and ankle behaviors during the movement and showed that the foot and ankle moved in a near circular fashion while the hip was maintained at about 180°. In a study of the movement on the novice and elite groups, Baudry et al. (2008) found greater differences of the foot-ankle 3D movements than the hip and shoulder joints between the groups. Baudry et al. (2009) further showed that the elite group had greater foot-ankle displacement in the horizontal plane than the notice group. Fujihara also studied the velocity changes of the center of mass in the horizontal plane (Fujhara, 2006; Fujihara et al., 2009). Our study is a pilot study that employed a single-subject design focusing on one elite athlete. We not only employed the traditional 3D kinematics approach to investigate the displacement characteristics of COM and foot, but also established the computer model developed in Lifemod, to examine its validity, and verify the simulation results with the experimental sEMG data. The results showed that the COG displacements during the movement cycle of the DLC in the Lifemod model were in generally agreement with the displacement outputs of the and the 3D motion analysis (Table 1). These results showed relatively accurate model formulation and validity. The results from the foot displacement (Table 1, Figures 1 and 2) of the model and 3D kinematics were also very close and consistent, and provided further evidence of the model’s accuracy. However, the COG displacement curves showed some minor differences in the displacement in the anterior, posterior, mediolateral and vertical directions between the two methods (Figure1 a and b), demonstrating the complexity of the human body and central nerve system, and the model did not account for some of the details of the system. It is important to enhance the model and examine its validity, in comparison with experimental data. Previous studies have shown that the movement trajectory of COG, shoulder, hip and foot-ankle are important variables that influence the stability of the DLC movement (Baudry et al., 2008; 2009; Fujhara, 2006; Fujihara et al., 2009; Grassi et al., 2005).

The DLC and other movements in gymnastics are among the most accurate and controlled human movements. The completion of every single movement requires participation and coordination of many skeletal muscles. The force magnitude, contraction mode, order, and duration of involved muscle groups have to be arranged and executed in a precise manner like a computer program under the command of central nervous system during movements. It is important to control and manipulate the timing of maximum muscle force output of certain muscles while sustaining prolonged and constant force outputs of other muscles. To accomplish these, an athlete needs to have great control of muscle contractions and coordination. Although computer modeling and simulation have been widely used in studies of human muscle forces and movements (Koo, 2005), research on DLC movement in pommel horse is still mostly limited to kinematics using experimental approaches. This study established the multi-body dynamics model for DLC using Lifemod software platform, examined the key movement characteristics in each movement phase, investigated the muscle actions of major muscle groups, and identified the muscles that had greater or longer force output during each movement phase. These results are important for further understanding of the movement and provide specific guidance of muscle strength training.

We compared the IEMG value computed from sEMG activities of the selected muscles to the muscle force changes estimated by the Lifemod model (Sun, 2007). The estimated muscle forces of triceps brachii and biceps brachii, latissimusdorsi and pectoralis major from the Lifemod model demonstrated patterns of agonist-antagonist coordination during the DLC movement cycle (Figures 1a and 1b). The peak value of triceps brachii was the highest among all tested muscles from the model outputs. The pectoralis major and biceps brachii showed a lower but longer-duration force outputs during most of the DLC cycle.

The outputs from the simulation demonstrated the force outputs throughout the four phases of the DLC in Figure 1a and 1b. During the phase 1 of the front double-support (T1), the results showed high force outputs of the triceps, latissimusdorsi, and deltoid muscles. These muscle activities supported the shoulder movements during the phase. The triceps brachii, latissimusdorsi and deltoid of both sides reached the first maximum in the phase 1. The left triceps and latissimusdorsi worked together to accomplish the desired movements of the shoulder in this phase. At the same time, the right biceps brachii and triceps brachii worked together in order to stabilize the shoulder joint to allow the latissimusdorsi to contract actively and provide the main source of power for the DLC phase. During this phase, the left arm pushed, while the left shoulder maintained an extended position, rotated internally, and abducted. Meanwhile the right shoulder joint adducted, rotated externally, and flexed to push off downwards from the pommel horse. The results of the iEMG showed high EMG activities from the left triceps braichii and latissimusdorsi, and left and right deltoids (100%, Figure 2a and 2b). The right triceps and latissimusdorsi, and the left deltoid muscle also reached values around 80%. These results supported the estimations of the model and showed that the triceps brachii, latissimusdorsi, and deltoid provided major force outputs in this DLC phase and the right triceps and biceps co-contracted.

In the phase 2, the left single support (T2), the left shoulder joint should have abducted and rotated internally while the right shoulder joint should have adducted to grab the handle aggressively. The simulation results showed that the left pectoralis major, right latissimusdorsi and pectoralis major provided major force outputs during the phase (Figure 1a and 1b). The pectoralis majors contracted continuously throughout the phase. The body was supported by the hand to prevent it from eccentric movement due to inertia effects. The iEMG results showed that the left pectoralis major reached 100% and the right latissimusdorsi and pectoralis major both reached relatively high activity levels (Figure 2a and 2b), which supported the force outputs of the model.

During the phase 3, the back double-support (T3), the right shoulder joint rotated internally and maintained abducted and the left hand pushed off promptly. The model estimated that the pectoralis major and biceps brachii of both sides showed continuous force outputs, and the right triceps and biceps co-contracted again to stabilize the shoulder joint (Figures 3 and 4). The EMG results showed high muscle activities of the right triceps and biceps during the phase, and both pectoralis majors maintained relatively high activities (Figure 2a and 2b), which further supported the force estimations of the model. In the final stage, the phase 4 of the right single-support (T4), the right arm abducted and rotated internally while the left shoulder joint adducted to grab the handle actively. The model simulation results showed high force outputs of the triceps brachii, latissimusdorsi, and deltoid muscles of both sides. These estimations were supported by the high EMG activities of the right triceps, pectoralis major, and latissimusdorsi, and the deltoid of both sides (Figure 2a and 2b).

To our knowledge, this is the first study examining muscle activities in the DLC, although Fujihara et al. (2009) designed an instrumented pommel horse to examine the reaction forces during the hand contact with the ring of the pommel horse. In future work, it is warranted to incorporate reaction force measurements into computer modeling and simulation work of the DLC movement to further improve accuracy of estimated muscle forces and joint dynamics.

In summary, the simulation and experimental results of COG and foot displacements showed good matches. The estimated muscle forces from the model were supported by the EMG data. These results showed a good validity of the model. The results of this study provided some evidence that may be beneficial for developing strength training plans of the upper extremity muscles that are specific to the movement. One of the applications of the model is to help identify errors in technique during training. It may be also possible to adjust muscle force related parameters in the model to seek optimal solutions of the movement in order to improve the technique. In future studies, the focus should be on developing athlete specific models to further examine effects of sequencing and muscle force peaks of involved muscles on movement dynamics of DLC.

## Figures and Tables

**Figure 1a:**
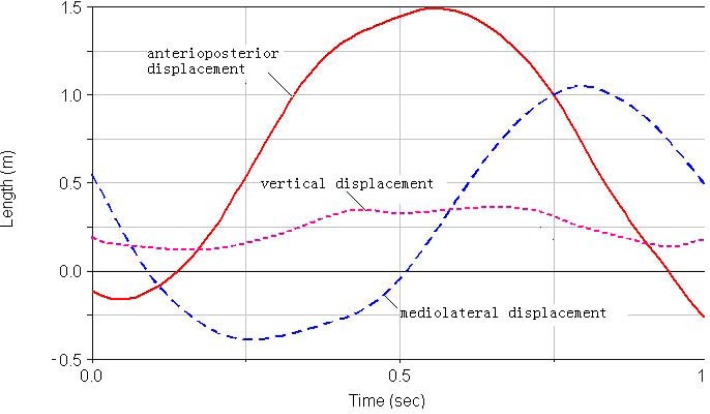
The curves for foot displacement in LifeMOD model

**Figure 1b:**
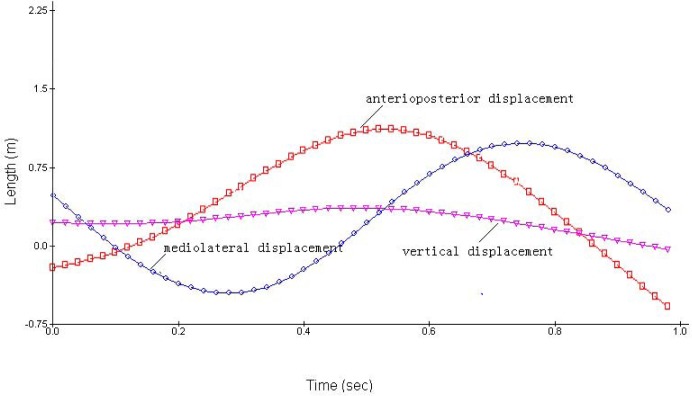
The curves for foot displacement in 3D motion analysis system

**Figure 2a:**
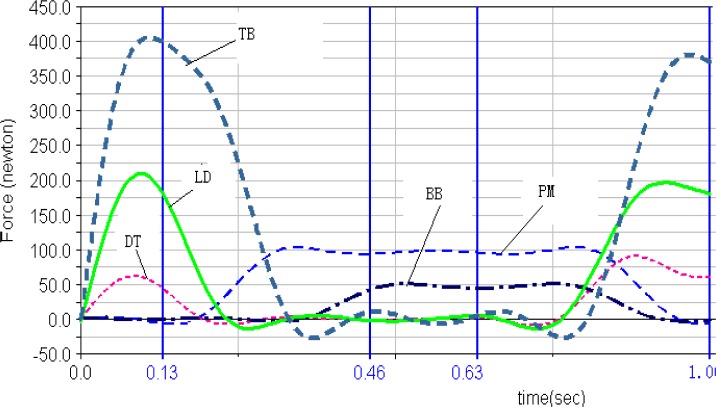
Left Pectoralis Major, Deltoid, Biceps Brachii,LatissimusDorsi and Triceps Brachii muscle forces in LifeMOD

**Figure 2b:**
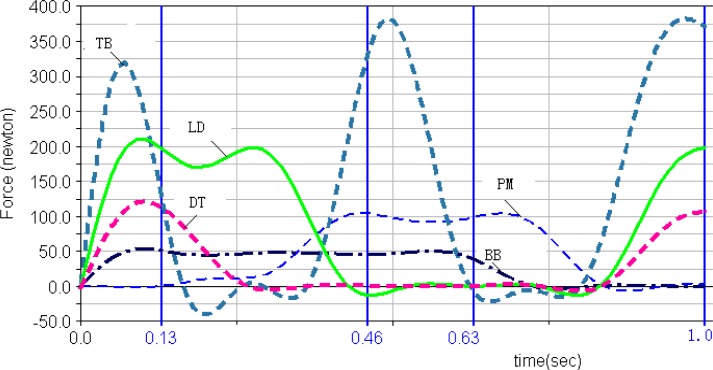
Right Pectoralis Major, Deltoid, Biceps Brachii, LatissimusDorsi and Triceps Brachii muscle forces in LifeMOD. The 4 vertical lines show the 4 phases

**Figure 3a:**
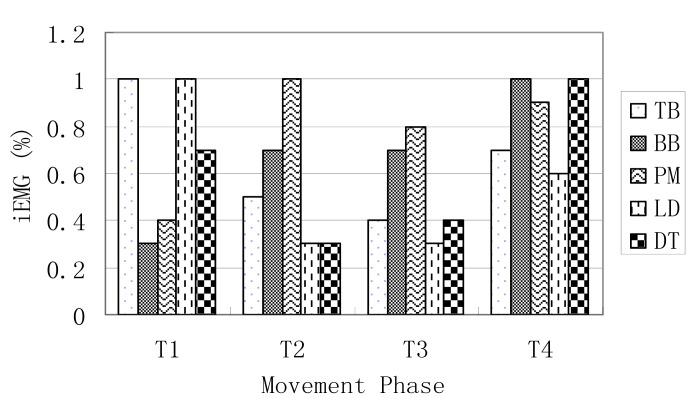
The iEMG values of the left -side muscles in forward double arm support (T1), left arm support (T2), backward double arm (T3), and right arm support (T4)

**Figure 3b:**
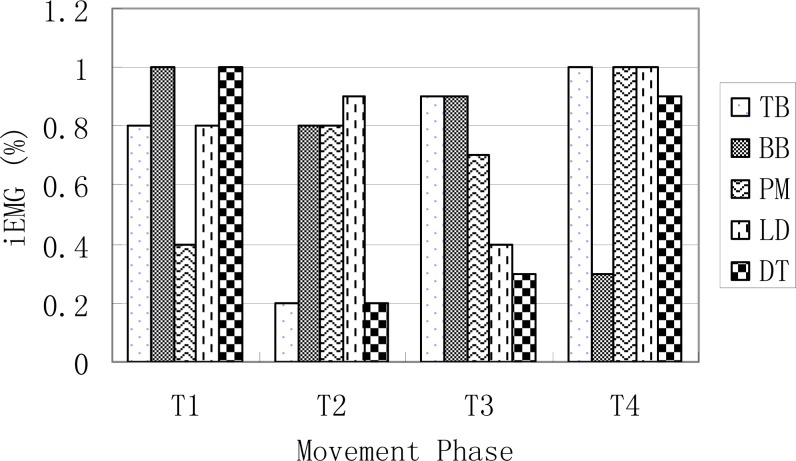
The iEMG values of the right side muscles in anterior double arm support (T1), left arm support (T2), posterior double arm (T3), and right arm support (T4)

**Table 1:** Experimental and model 3D and foot displacement of COG

		Mediolateral (m)	Vertical (m)	Anterioposterior (m)
COG	3D	0.13	0.14	0.19
	LifeMOD	0.13	0.14	0.17
Foot	3D	1.48	0.15	1.91
	LifeMOD	1.5	0.17	1.82
